# Laboratory Reporting of *Staphylococcus aureus* with Reduced Susceptibility to Vancomycin in United States Department of Veterans Affairs Facilities^1^

**DOI:** 10.3201/eid0804.010245

**Published:** 2002-04

**Authors:** Stephen M. Kralovic, Linda H. Danko, Gary A. Roselle

**Affiliations:** * Department of Veterans Affairs Central Office, Washington, D.C., USA; † Cincinnati Veterans Affairs Medical Center, Cincinnati, Ohio, USA; ‡ University of Cincinnati College of Medicine, Cincinnati, Ohio, USA

**Keywords:** surveillance, veterans, communicable diseases, vancomycin resistance, staphylococcus, emerging diseases

## Abstract

A national survey was sent to all appropriate Veterans Health Administration (VA) medical facilities asking abut the ability to test for *Staphylococcus aureus* with reduced susceptibility to vancomycin (SARV) (MICs >4 μg/mL). Also, a request was made for the number of patients having SARV isolated during a 1-year period. Nineteen patients from eight sites across the country had isolation of SARV. Of these, MicroScan (Dade Behring, Inc, MicroScan Division, West Sacramento, CA) technology was used for 17 patients, Vitek (Hazelwood, MO) was used for 1 of the remaining 2 patients, and E-test (AB Biiodisk North America, Inc, Piscataway, NJ) for the other. All patients with this organism had microbiology testing done onsite in the reporting VA facility’s College of American Pathologists-approved laboratory. For comparison, similar data were obtained for a 1-year period 2 years prior to the current survey; seven patients from four sites were verified to have a SARV. Between the two survey periods the reported cases of SARV increased 170%, indicating a need for continued surveillance and potentially a need to initiate a collection of isolates for further analysis.

Emerging microbial resistance is a substantial threat to health (*1*). With the discovery of methicillin-resistant *Staphylococcus aureus* (MRSA) that also had intermediate resistance to vancomycin in 1996 in Japan, more intense scrutiny has been given to identifying resistance and reduced susceptibility in staphylococcal species (*2*–*4*). Even before the Japanese isolate was identified, in vitro evidence that vancomycin-resistant enterococci (VRE) could transfer resistance to staphylococci led to concern for spread of vancomycin resistance to the staphylococci (*5*–*7*).

In 1995, the Centers for Disease Control and Prevention (CDC) recommended microbiology laboratories be vigilant for the occurrence of vancomycin resistance in staphylococci along with confirmatory testing and reporting such resistance to public health authorities (*5*). Further, recommendations in 1997 called for vigilance for reduced susceptibility to vancomycin (MIC >4 μg/mL) rather than just vancomycin resistance (MIC >32 μg/mL) (*8*,*9*). These recommendations included awareness of the significance of isolates with reduced susceptibility, confirmatory testing of suspect isolates, retesting staphylococci isolated from patients who have failed to respond to vancomycin therapy, and notification of public health authorities. The National Committee for Clinical Laboratory Standards (NCCLS) has set *S. aureus* breakpoints for vancomycin at <4 μg/mL is interpreted as susceptible, 8-16 μg/mL is intermediate and >32 μg/mL is resistant (*10*). Despite the fact that an MIC = 4 μg/mL is defined as susceptible by NCCLS standards, it is considered to be at the borderline of resistance (*11*). In particular, *S. aureus* strains that are methicillin or oxacillin resistant and that have an MIC to vancomycin of ≥4 μg/mL should be suspected for decreased susceptibility to vancomycin and should be considered for additional testing strategies because of the possible subpopulation heterogeneity of *S. aureus* isolates with these MIC results (*11*,*12*).

Recent studies from CDC indicate that proper identification of antibiotic resistance may be difficult despite adequate capacity for testing (*13*). A selected survey of laboratories participating in CDC surveillance (Active Bacterial Core Surveillance and Emerging Infections Programs Network) indicates that these issues may occur despite active participation in CDC activities (*13*). A more recent study involving the worldwide WHONET users suggested that these difficulties in identification of antibiotic resistance might be even greater (*14*). These studies indicate that real-world application of recommended standards into typical day-to-day functioning does not mimic the functioning and results seen in tightly controlled study situations.

Through its annual survey for federal fiscal year (FY) 1999 (October 1, 1998 through September 30, 1999), the Infectious Diseases Program Office of the United States Department of Veterans Affairs (VA) undertook a national assessment of the VA health-care system surveillance for SARV. In addition to identifying cases with vancomycin (glycopeptide)-resistant or -intermediate isolates, we sought vancomycin-, glycopeptide-resistant, or intermediate isolates and sought to identify those cases with the potential for decreased susceptibility to vancomycin. Therefore, we have chosen to use the designation of SARV to encompass all of these. During FY 1999, the VA health-care system served a population of >3.6 million persons in its 172 medical centers and >600 outpatient clinics; it had approximately 600,000 inpatient discharges and >35 million outpatient visits during that same period.

## Materials and Methods

Annually since 1990, the Infectious Diseases Program Office for VA Central Office has distributed an Infectious Diseases/Infection Control survey to all VA medical center reporting sites across the country that request data on several topics. The process for the survey begins with the distribution of the annual survey instrument (questionnaire) to each VA medical center reporting site; this is delivered to the administrator responsible for the facility. Subsequently, a 2-week period is established for receipt of responses to the survey. Responses are made by electronic entry into a central database by each site. Each site notes a point of contact for subsequent data verification. After the 2-week period, the ability to access the database for entry is closed to the medical center reporting sites.

Administrative review by the Infectious Diseases Program Office identifies medical centers that have omitted data. The point of contact is queried (either by telephone or e-mail) as to the nature of the omission. Concomitant with the review of data for omission, a preliminary analysis of the submitted data is undertaken to assess for accuracy of other reported data and consistency of reporting with previously submitted data from the medical center. There are several questions in the Infectious Diseases/Infection Control annual survey that serve as controls for analysis. If there is concern that the submitted data may be inaccurate, the point of contact for the site is also queried to verify these data.

Beginning in 1998 (for FY 1997 data), the survey included two questions regarding SARV. The following questions were asked, 1) Does your facility do or obtain testing to identify reduced susceptibility to vancomycin (MIC >4 μg/mL) for *Staphylococcus aureus*? Yes or no? 2) If yes, report the number of patients (not cultures) with *Staphylococcus aureus* with reduced susceptibility to vancomycin (MIC >4 μg/mL).

Any site reporting presence of a patient with SARV was contacted by the Infectious Diseases Program Office to verify accuracy of the report for both FY 1997 and 1999 data. During the contact by the Infectious Diseases Program Office (January 2001) for FY 1999 data, additional information was requested from those sites that reported and verified patients with SARV. This additional information included verification that the isolate was indeed *S. aureus*, identification of the susceptibility testing methods, source of the specimen, inpatient or outpatient status at the time of specimen acquisition, and MIC to vancomycin. Query was also made regarding confirmatory testing of vancomycin susceptibility of the patient isolate, susceptibility testing to other antimicrobial agents, and current availability of the isolate.

## Results

For FY 1997, there was 100% response to the survey instrument (146 reporting sites), although not all questions were completed. Initially 11 sites reported 284 patients with SARV. After contact and verification of the survey results by the Infectious Diseases Program Office with these sites, seven patients were reported to have SARV from four of the sites. Rationale for discounting initially reported cases after verification included misinterpretation of the question to be requesting information on VRE or misinterpretation of the question to mean MRSA.

For FY 1999, there was 99% response to the survey instrument (142 of 143 reporting sites), though not all questions were initially completed. With regard to the ability of the reporting facility to do or obtain testing to identify SARV, 142 reporting sites answered this question, with 123 (86%) of the sites responding “yes.” Of the 123 sites reporting yes, initially 13 sites reported 195 patients with SARV. After contact and verification by the Infectious Diseases Program Office, the number of verified, reported cases was revised to eight sites reporting 19 patients with SARV. Reasons for change of reported numbers to verified numbers included misinterpretation of the question to mean MRSA as well as one isolate with a difficult determination by the original MIC method used (reporting an MIC >16 μg/mL) but with confirmatory testing defining an MIC = 1.5 μg/mL. Microbiology testing was noted to be done onsite in a CAP-approved laboratory for all reported and verified cases.

The specimen sources for these isolates were five from tissue or wounds, five from a urinary source, four from sputum, two from abdominal or peritoneal sources, and one each from blood, eye, and synovial fluid. Initial susceptibility testing showed 17 used MicroScan technologies (Dade Behring, Inc, MicroScan Division, West Sacramento, CA) and one each of bioMerieux Vitek (Hazelwood, MO), and E-test (AB Biiodisk North America, Inc, Piscataway, NJ) (Table 1). Confirmatory testing was done on only 2 of the 19 reported cases, using E-test and MicroScan technology (Table 1). One isolate was sent to CDC for confirmation. However, as noted above, confirmatory testing had also been done on at least one occasion to refute presence of SARV. Sixteen of the isolates were reported to have an MIC = 4 μg/mL, one was reported to have an MIC = 8 μg/mL (noted to be an intermediate sensitivity interpretation), one was reported at >16 μg/mL, while one was reported at >32 μg/mL; these last two isolates were interpreted as being resistant. Six of these specimens were obtained from patients during an outpatient encounter, while seven were obtained while patients were on inpatient status, and five were from patients in a nursing home. Only one of the 19 case isolates had been stored and is available for further analysis.

**Table 1:** Information from reported isolates of *Staphylococcus aureus* with reduced susceptibility to vancomycin from United States Veterans Health Administration medical facilities, FFY^a^ 1999

Case	Inpt/Outpt/NH^b^	Specimen	Method^c^ (Instrumentation/panel)	MIC/susceptibility^d^	Confirmation of susceptibility	Confirmation methodology
1	Outpt	Ear tissue	MicroScan Walkaway version 22.01/Gram Pos Combo Panel 10	> 16 μg/mL/R	Yes	MicroScan Walkaway
2	Inpt/SICU^e^	ABD^f^ and VP^g^ shunt	MicroScan Walkaway version 22.06/Pos Combo 12	= 4 μg/mL/S	No	--
3	Inpt	Sputum	MicroScan Walkaway version 22.26/Pos Combo 14	= 4 μg/mL/S	No	--
4	Outpt	Urine	MicroScan Walkaway version 22.06/Pos Combo 12	= 4 μg/mL/S	No	--
5	Outpt	Leg	MicroScan Walkaway/Pos Combo Panel 10	= 4 μg/mL/S	No	--
6	Outpt	Eye	MicroScan Walkaway/Pos Combo Panel 10	= 8 μg/mL/(I)	No	--
7	Inpt	Peritoneal	MicroScan Walkaway/Pos Combo Panel 10	= 4 μg/mL/S	No	--
8	Inpt	Urine	MicroScan Walkaway/Pos Combo Panel 10	= 4 μg/mL/S	No	--
9	Outpt	Wound	MicroScan AutoScan/Pos Combo 11	= 4 μg/mL/S	No	--
10	NH	Sputum	MicroScan AutoScan/Pos Combo 11	= 4 μg/mL/S	No	--
11	NH	Sputum	MicroScan AutoScan/Pos Combo 11	= 4 μg/mL/S	No	--
12	NH	Urine	MicroScan AutoScan/Pos Combo 11	= 4 μg/mL/S	No	--
13	NH	Urine	MicroScan AutoScan/Pos Combo 11	= 4 μg/mL/S	No	--
14	NH	Wound	MicroScan AutoScan/Pos Combo 11	= 4 μg/mL/S	No	--
15	Inpt	Foot wound	E-test; VCN^h^ screen plate	= 4 μg/mL/S	Yes	Sent to CDC^i^; E-test
16	Outpt	Synovial fluid	MicroScan Walkaway/Gram Pos Combo Panel 10	= 4 μg/mL/S	No	--
17	Inpt/MICU^j^	Sputum	MicroScan Walkaway/Gram Pos Combo Panel 10	= 4 μg/mL/S	No	--
18	Inpt/ICU^k^	Blood	MicroScan AutoScan version 22.01/Pos Combo Panel 10	= 4 μg/mL/S	No	--
19	Inpt/MICU	Urine	BioMerieux Vitek VTK-R version 07.01/GPS-102	> 32 μg/mL/R	Unable to determine	--

^a^FFY=Federal fiscal year. ^b^Inpt=inpatient; Outpt=outpatient; NH=nursing home. ^c^ bioMerieux Vitek (Hazelwood, MO), E-test (AB Biodisk North America, Inc., Piscataway, NJ), MicroScan (Dade Behring Inc., MicroScan Division, West Sacramento, CA). Where data were available, software version of technology provided. All MicroScan methods used conventional 24-hour incubation susceptibility panels. ^d^ Susceptibility interpretation at the reporting site (S=sensitive, I=intermediate, R=resistant). ^e^ SICU=surgical intensive care unit. ^f^ABD=abdominal. ^g^VP=Ventriculo-peritoneal. ^h^VCN=vancomycin. ^I^CDC=Centers for Diseases Control and Prevention. ^j^MICU=medical intensive care unit. ^k^ICU=intensive care unit.

Reported susceptibility testing to other antimicrobial agents are noted in Table 2 and Table 3 where data were available; not all isolates had susceptibility testing done against all antimicrobial agents reported. Beta-lactamase activity was present for 14 of the 16 isolates. For penicillin-type antibiotics, 12 of the 19 isolates had oxacillin resistance. There was also a relatively high degree of resistance to the cephalosporins tested (data not shown) and six of eight isolates were resistant to imipenem. Antibiotic susceptibility testing against other agents used to treat gram-positive infections showed varying degrees of resistance, with 4 of 15 isolates resistant to rifampin, 14 of 18 resistant to erythromycin, 11 of 17 resistant to clindamycin, 4 of 17 resistant to tetracycline, only 1 of the 19 isolates resistant to trimethoprim/sulfamethoxazole, and none of 4 isolates resistant to chloramphenicol. For the quinolones and other agents, 5 of 16 were resistant to ciprofloxacin, 4 of 5 were resistant to levafloxacin, 4 of 7 were resistant to ofloxacin, and 3 of 16 were resistant to gentamicin.

**Table 2:** Susceptibility to selected gram-positive agents of isolates of reported *Staphylococcus aureus* with reduced susceptibility to vancomycin from United States Veterans Health Administration medical facilities, FFY 1999^a^

Case	Beta-lactamase	MIS/sensitivity status							
		oxacillin	imipenem	rifampin	TMP/SMX	erythromycin	TCN	clindamycin	chloramphenicol
1	negative	/R			/S	/R	/S	/R	
2	positive	<0.5 μg/mL	<4 μg/mL	<1 μg/mL	<2 μg/mL	=0.5 μg/mL	<2 μg/mL	<0.25 μg/mL	
3	positive	>2 μg/mL	>8 μg/mL/R	<1 μg/mL	<2 μg/mL	>4 μg/mL/R	<2 μg/mL	>2 μg/mL/R	
4	positive	>4 μg/mL	>8 μg/mL/R	<1 μg/mL	<2 μg/mL	>4 μg/mL/R	<2 μg/mL	>2 μg/mL/R	
5	positive	<0.5 μg/mL/S	>8 μg/mL/R	>2 μg/mL/R	<2/38 μg/mL/S		>8 μg/mL/R	>2 μg/mL/R	
6	positive	>2 μg/mL/R	2 μg/mL/R	>1 μg/mL/S	<2/38 μg/mL/S	>4 μg.mL/R	<2 μg/mL/S		8 μg/mL/S
7	positive	>2 μg/mL/R	>8 μg/mL/R	>2 μg/mL/R	>2/38 μg/mL/R	>4 μg/mL/R	>8 μg/mL/R		16 μg/mL/I
8	positive	>2 μg/mL/R	>8 μg/mL/R	>1 μg/mL/S	<2/38 μg/mL/S	>4 μg/mL/R	<2 μg/mL/S	>2 μg/mL/R	16 μg/mL/I
9	positive	>2 μg/mL/R		<1 μg/mL/S	<2 μg/mL/S	>4 μg/mL/R	<2 μg/mL/S	>2 μg/mL/R	
10	negative	<0.5 μg/mL/S		<1 μg/mL/S	<2 μg/mL/S	<0.25 μg/mL/S	<2 μg/mL/S	=0.5 μg/mL/S	
11	positive	<0.5 μg/mL/S		<1 μg/mL/S	<2 μg/mL/S	>4 μg/mL/R	<2 μg/mL/S	<0.25 μg/mL/S	
12	positive	>2 μg/mL/R		<1 μg/mL/S	<2 μg/mL/S	>4 μg/mL/R	<2 μg/mL/S	>2 μg/mL/R	
13	positive	<0.5 μg/mL/S		<1 μg/mL/S	<2 μg/mL/S	>4 μg/mL/R	>8 μg/mL/R	>2 μg/mL/R	
14	positive	>2 μg/mL/R		>2 μg/mL/R	<2 μg/mL/S	>4 μg/mL/R	<2 μg/mL/S	>2 μg/mL/R	
15		/R			<2 μg/mL/S	/R	<2 μg/mL/S	<2 μg/mL/S	=8 μg/mL/S
16		1 μg/mL/S			<2/38 μg/mL/S	0.5 μg/mL/S		>2 μg/mL/R	
17		>4 μg/mL/R			<2/38 μg/mL/S	>4 μg/mL/R		>2 μg/mL/R	
18	positive	<0.05 μg/mL	<4 μg/mL	<1 μg/mL	<2 μg/mL	<0.25 μg/mL	<2 μg/mL	0.5 μg/mL	
19	positive	>8 μg/mL/R		>4 μg/mL/R	<16 μg/mL/S	>8 μg/mL/R	>16 μg/mL/R	>8 μg/mL/R	
Total^b^	14/16=pos	12/19 =R	6/8=R	4/15=R	1/19=R	14/18=R	4/17=R	12/17=R	0/4=R

^a^FFY=Federal fiscal year; S=susceptible, I=intermediate and R=resistant based on laboratory interpretative criteria. ^b^The authors took the liberty of placing interpretation on some reported MIC values that did not have an interpretation of S, I, or R on information provided from the facility.

**Table 3:** Reported susceptibility to quinolones and other agents of reported isolates of *Staphylococcus aureus* with reduced susceptibility to vancomycin from United States Veterans Health Administration medical facilities, FFY 1999^a^

Case	MIC/sensitivity status					
	ciprofloxacin	levofloxacin	ofloxacin	norfloxacin	gentamicin	furodantin
1	/S					
2	<1 μg/mL			<4 μg/mL	<1 μg/mL	<32 μg/mL
3	>2 μg/mL/R	>4 μg/mL/R		>8 μg/mL	<1 μg/mL	<32 μg/mL
4	>2 μg/mL/R			>8 μg/mL	<1 μg/mL	<32 μg/mL
5	<1 μg/mL/S	4 μg/mL/I			<1 μg/mL/S	
6	>2 μg/mL/R	>4 μg/mL/R			8 μg/mL/I	
7	>2 μg/mL/R	>4 μg/mL/R			>8 μg/mL/R	
8	>2 μg/mL/R	>4 μg/mL/R			>8 μg/mL/R	
9	>2 μg/mL/R		>4 μg/mL/R		<1 μg/mL/S	<32 μg/mL
10	=2 μg/mL/I		<2 μg/mL/S		<1 μg/mL/S	=64 μg/mL
11	>2 μg/mL/R		>4 μg/mL/R		<1 μg/mL/S	<32 μg/mL
12	>2 μg/mL/R		>4 μg/mL/R		<1 μg/mL/S	<32 μg/mL/S
13	>2 μg/mL/R		>4 μg/mL/R		<1 μg/mL/S	<32 μg/mL/S
14	<1 μg/mL/S		<2 μg/mL/S		<1 μg/mL/S	<32 μg/mL
15					/R	
16						
17						
18	<1 μg/mL				<1 μg/mL	
19	<0.5 μg/mL/S		<1 μg/mL/S		<2 μg/mL/S	
Total	9/16=R	4/5=R	4/7=R		3/16=R	

^a^FFY=Federal fiscal year; S=susceptible, I=intermediate and R=resistant based on laboratory interpretative criteria.

## Discussion

In this survey, patients were reported to have SARV in the VA system for both FYs 1997 and 1999. Two VA sites reported cases of SARV in both surveys. Comparison of the two surveys indicates an increase of 170% in the number of cases reported in 1999 compared with 1997. This finding is in contradistinction to information reported by MRL Pharmaceutical Services, where none of 3,797 *S. aureus* isolates had reduced susceptibility to vancomycin in 1999 (*15*). Even though different methods of data accrual were used, both surveys rely on NCCLS-based susceptibility criteria (*10*). Despite the fact that presence of SARV appears to be a low-incidence occurrence at this time, the reason for different occurrences of SARV between the VA and this other national data set (*15*) is not readily apparent from the data present. The difference of findings, even though both use NCCLS-based susceptibility criteria, may be based on the fact that the MRL study used one consistent microbroth dilution method for susceptibility testing whereas our population-based reporting survey encompasses numerous susceptibility testing methods (MicroScan, Vitek, E-test, screening plates) more analogous to real-world application of technologies. Tenover et al. (*11*) demonstrated different methods of susceptibility testing (e.g., MicroScan Rapid panels and disk diffusion) have been shown to be unreliable in detecting *S. aureus* strains with reduced susceptibility of vancomycin; none of the VA laboratories reporting SARV used those methods considered to be unreliable (Table 1). Sampling size may be a factor, as we do not have the total number of isolated tests nationwide in the VA, but the total number likely exceeds the number of isolates in the MRL study. Different populations sampled or the recent CDC studies indicating difficulty in delimiting antibiotic resistance (*13*,*14*) might all contribute to this difference as well.

Because of limitations in our survey methods, we are unable to supply information on the total number of *S. aureus* isolates (or persons with *S. aureus* isolated) within the VA system nationwide, from which to determine prevalence estimates for comparative purposes to other studies (*16*,*17*). Data from Wilcox et al. and Aucken et al. indicate that about 15% of isolates in the United Kingdom had a vancomycin MIC of 4 μg/mL on initial testing (*16*,*17*). These two reports also indicate a low prevalence of vancomycin (glycopeptide)-intermediate or -resistant isolates upon confirmation. The reports also lend support to the finding that susceptibility testing of *S. aureus* to vancomycin by disk diffusion, which is commonly used in the United Kingdom, is not as reliable as other methods of testing for reduced susceptibility of *S. aureus* to vancomycin (*11*).

For each of the 2 years surveyed, more sites initially reported patients with SARV than were present after verification by the Infectious Diseases Program Office. The most common reason noted for the inaccurately reported data was misinterpretation of the question to mean MRSA, indicating that despite a simply worded question giving specific definitions, data validation is important. Validation is especially important for low-incidence diseases, for which a few misreported cases may significantly alter the final outcome.

Despite CDC recommendations on confirmatory testing of suspect isolates, repeat susceptibility testing is not being performed consistently for all isolates. If confirmatory testing is being performed, it is not being recorded; therefore, it is not reported in this retrospective review. NCCLS does not indicate the need for repeat testing (*10*). Further, from the survey we were not able to determine if confirmatory testing of the isolate to indeed be *S. aureus* was occurring. However, each site did note that it was confident of the organism identification. Perhaps of greater importance is the recognition that despite CDC recommendations for confirmatory testing to be done for isolates of staphylococci with MICs >4 μg/mL to vancomycin, the Infectious Diseases Program Office was informed by several of the sites reporting SARV that an MIC of 4 μg/mL was interpreted as susceptible by NCCLS criteria; (*10*) therefore, it was not necessary to confirm this result. The lack of confirmatory testing indicates poor recognition for the significance of *S. aureus* having the potential for reduced susceptibility to vancomycin. Also, only one site (of eight sites in FY 1999) reported contacting a public health authority about a SARV isolate (i.e., sent to CDC for confirmatory testing), again indicating a lack of recognition of important Public Health Service recommendations. No other reports indicate the extent of adherence to such national recommendations about SARV. Even though this lack of recognition and reporting is not the same as the capacity to detect antimicrobial resistance, it is analogous to CDC findings that confirmation of susceptibility for suspected SARV was as low as 39% (*13*).

Upon review of other antimicrobial susceptibilities of the 19 SARV cases from FY 1999, a high degree of resistance to other agents was found; however, a third of isolates were noted to be susceptible to oxacillin or methicillin. Therefore, not all reported cases of SARV were also MRSA. Non-*aureus* staphylococcal species were not reviewed in this survey. Some reports indicate that coagulase-negative staphylococci also have reduced susceptibility to vancomycin (*18*).

Given the limitation to two basic questions imposed by the Infectious Diseases/Infection Control annual survey methods, the full extent and characterization of *S. aureus* with reduced susceptibility to vancomycin in the VA cannot be accurately assessed. However, this method has indicated that SARV exists in the VA health-care system, the occurrence of which has increased between the 2 years reviewed. This study also identified the importance of data validation as evidenced by misinterpretation of clearly stated and defined questions. With the presence of this organism and its apparent increase in occurrence, continued surveillance is indicated. A more thorough analysis of the extent and characteristics of this organism in the VA system would be beneficial to both VA and public health in general; this analysis might include patient characteristics, antibiotic use in patients before acquisition of SARV, and collection and storage of isolates for further laboratory analysis. Along with further characterization of the epidemiology of this organism, increasing awareness as to the significance of SARV is indicated.
